# Acute Tubulointerstitial Nephritis due to Human Papillomavirus Vaccination

**DOI:** 10.31662/jmaj.2023-0096

**Published:** 2023-11-27

**Authors:** Sachiko Nakaoka, Shinichi Tsubata, Yuichi Adachi

**Affiliations:** 1 Department of Pediatrics, Toyama Red Cross Hospital, Toyama, Japan; 2 Pediatric Allergy Center, Toyama Red Cross Hospital, Toyama, Japan

**Keywords:** acute tubulointerstitial nephritis, clinically mild encephalitis/encephalopathy with a reversible splenial lesion, human papillomavirus vaccine, drug lymphocyte stimulation test

## Abstract

Acute tubulointerstitial nephritis (ATIN), a rare cause of acute kidney injury in children, is caused by various factors such as drugs, infection, and systemic inflammation. We herein present a case of ATIN with mild encephalitis/encephalopathy with reversible splenial lesion (MERS)-like findings on head magnetic resonance imaging (MRI), which was associated with human papillomavirus (HPV) vaccination. A 14-year-old girl presented to our hospital with a high fever for 5 days. Results of common laboratory tests were normal except for increased C-reactive protein (CRP) levels and erythrocyte sedimentation rate (ESR). Antibiotics were administered, and the fever promptly resolved after admission. After 7 weeks, she was readmitted due to a high fever for 4 days. In addition to increased CRP levels and ESR, urine test revealed high urine *N*-acetyl-β-D-glucosaminidase and β-2-microglobulin levels, and a renal scintigram showed mild bilateral uptake of 67Ga-citrate, consistent with the pathology of ATIN. Furthermore, head MRI, which was performed because the patient experienced prolonged headaches, revealed MERS-like lesions, although she did not have other neurological symptoms. Detailed examination of her medical records revealed that she had developed high fever 10 days after the third HPV vaccination and another previous episode of high fever 12 weeks after the second HPV vaccination. Based on these findings, we concluded that the ATIN and MERS-like lesions could have been associated with HPV vaccination. Although HPV vaccination is important for preventing uterine cancer, physicians must be vigilant about its various potential adverse effects, including ATIN.

## Introduction

Vaccination against human papillomavirus (HPV) is important for cancer prevention ^(1)^. However, some adverse effects of HPV vaccination, including headache, general fatigue, complex regional pain syndrome, and acute disseminated encephalomyelitis, have been reported ^(2)^. Acute tubulointerstitial nephritis (ATIN), a rare cause of acute kidney injury, can lead to chronic kidney disease in children ^(3)^. The leading causes of ATIN can be classified as drug-related, infectious, systemic, autoimmune, genetic, and idiopathic, including tubulointerstitial nephritis and uveitis syndrome (TINU) and anti-tubular basement membrane disease ^(3), (4)^. Regardless of the cause, most patients with ATIN present with nonspecific symptoms or acute kidney function worsening ^(3)^. We herein present a case of ATIN and mild encephalitis/encephalopathy with reversible splenial lesion (MERS)-like findings on head magnetic resonance imaging (MRI) that were associated with HPV vaccination.

## Case Report

A previously healthy 14-year-old girl presented to our institution with high fever and complaints of anorexia and malaise. The results of physical examination were normal, and the laboratory findings were as follows: white blood cell (WBC), 5000/μL; C-reactive protein, 3.08 mg/dL; serum blood urea nitrogen (BUN), 8 mg/dL; serum creatinine, 0.54 mg/dL (estimated glomerular filtration rate [e-GFR], 98.7 mL/min/1.73 m^2^). Two days before admission, she was administered β-lactam antibiotics, and then after admission, she was administered macrolide antibiotics. One day after admission, her fever resolved, and she was discharged 5 days later. The cause of her fever could not be identified during the initial admission.

**Figure 1.**
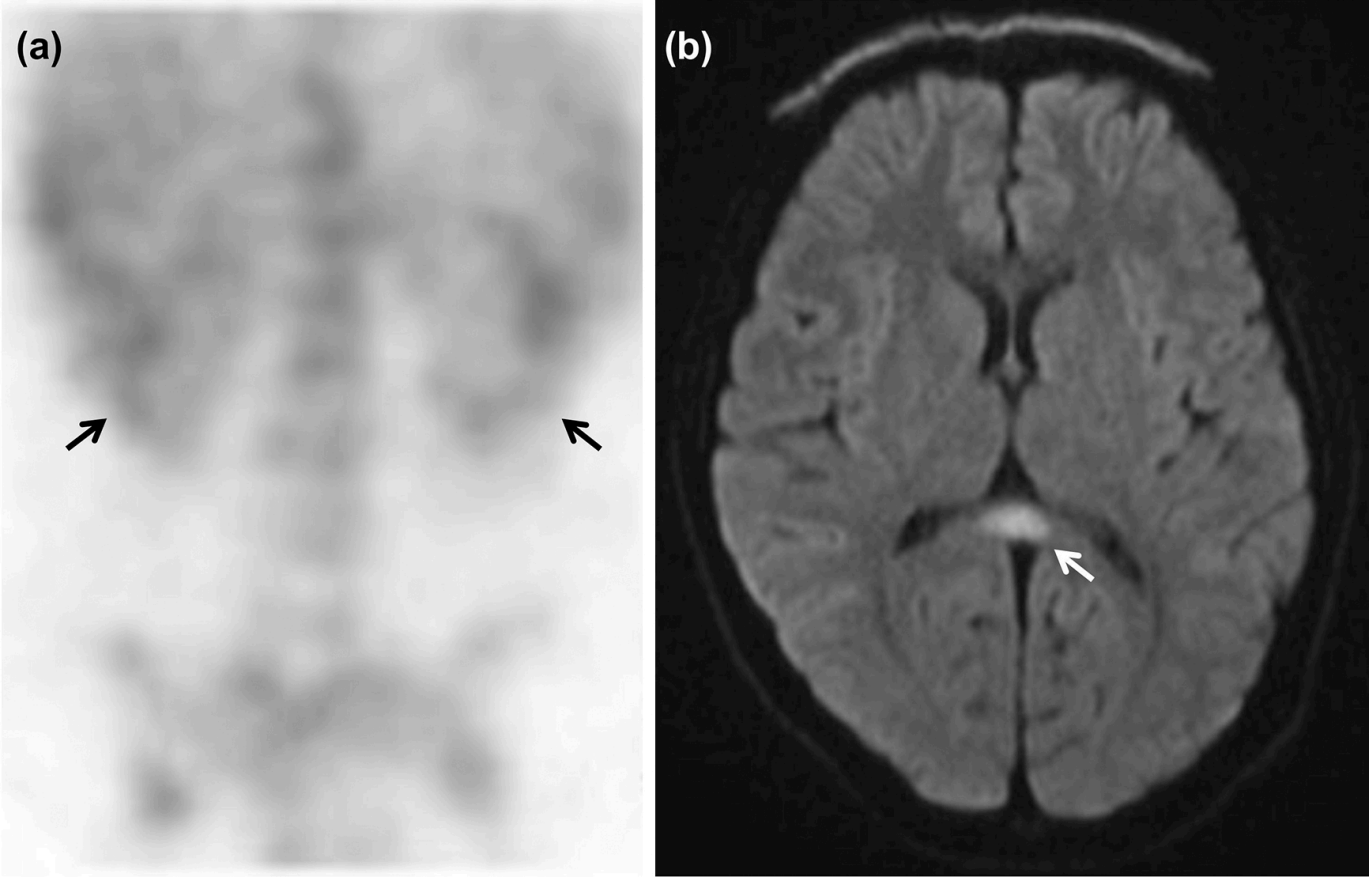
Results of 67Ga-citrate scintigraphy and brain magnetic resonance imaging (MRI). (a) The result of 67Ga-citrate scintigraphy indicates mild bilateral renal uptake (black arrows). (b) Axial MRI images. Diffusion-weighted images show a hyperintense area in the splenium of the corpus callosum (white arrow), which completely disappeared at the follow-up MRI.

Seven weeks after admission, she was readmitted to our hospital with high fever. The results of the general examination were normal, and the laboratory findings were as follows: WBC, 5500/μL; C-reactive protein, 7.17 mg/dL; serum BUN, 10 mg/dL; serum creatinine, 0.60 mg/dL (eGFR 89.2 mL/min/1.73 m^2^). In addition, the results of the urinalysis were as follows: urine *N*-acetyl-β-D-glucosaminidase (NAG), 14.7 IU/L; urine β-2-microglobulin (β2MG), 7139 μg/L. Accordingly, renal tubular disorder with mild renal dysfunction was considered. Mild bilateral renal uptake was observed on 67Ga-citrate scintigraphy (Figure 1a), confirming the diagnosis of ATIN. Ophthalmological examination revealed no findings of uveitis. The patient also complained of prolonged headaches; however, she had normal consciousness and blood pressure. Head MRI revealed MERS-like lesions in the splenium of the corpus callosum (Figure 1b). Her fever subsided on the 6th day of hospitalization without any treatment. Furthermore, the levels of urine NAG and β2MG decreased and the eGFR recovered to 108.5 mL/min/1.73 m^2^. Detailed examination of her medical records revealed that she had developed high fever 10 days after the third HPV vaccination and another previous episode of high fever 12 weeks after the second HPV vaccination. A drug lymphocyte stimulation test (DLST) confirmed that the patient had a stimulation index of 5352% for the HPV vaccine (cutoff value for DLST positivity, 180%). Finally, we diagnosed her with ATIN and MERS-like lesions associated with HPV vaccination.

## Discussion

ATIN most commonly occurs due to drug exposure ^(3)^; however, the diagnosis of drug-induced ATIN is challenging. Although drug-induced ATIN often occurs less than 2 weeks after drug exposure, the disease onset period can range from days to months depending on the drug ^(5)^. Evidence for HPV vaccine-induced ATIN is limited. To date, only two cases of HPV vaccine-associated TINU have been reported ^(6)^. The patient in this report did not take any medications other than HPV vaccines before these episodes. The interval between vaccination and symptom onset was shorter for the third vaccination than for the second one. DLST, which measures drug-induced T-cell proliferation in vitro, is a useful diagnostic test for various types of drug hypersensitivity ^(5)^. Because vaccines activate immunity, the DLST results for vaccines should be interpreted with caution.

The patient also experienced prolonged headaches, and brain MRI revealed MERS-like lesions. MERS is a clinicoradiological syndrome characterized by acute encephalopathy and an ovoid lesion in the mid-splenium of the corpus callosum on MRI ^(7)^. Infectious triggers are identified in approximately half of the cases, and a case of MERS after mumps vaccination has been reported ^(7)^. Although the pathophysiology of the syndrome is not well understood, hypersensitivity to the HPV vaccine may be involved in the pathological mechanism.

In conclusion, we report the case of a girl with ATIN and MERS-like lesions associated with HPV vaccination. This case highlights an extremely rare complication of HPV vaccination that provides important information for physicians. HPV is the most common cancer-causing pathogen in women ^(1)^. HPV vaccination is effective in preventing benign and malignant conditions ^(1)^. While our report does not deny the efficacy of the HPV vaccine, physicians should be vigilant of its potential side effects.

## Article Information

### Conflicts of Interest

None

### Author Contributions

All authors met the ICMJE authorship criteria.

### Informed Consent

Written informed consent was obtained from the patient and her guardians for the publication of this case report and any accompanying images.
